# The Influence of Entrepreneurs’ Online Popularity and Interaction Behaviors on Individual Investors’ Psychological Perception: Evidence From the Peer-To-Peer Lending Market

**DOI:** 10.3389/fpsyg.2022.825478

**Published:** 2022-02-18

**Authors:** Jiaji An, He Di, Guoliang Liu

**Affiliations:** School of Management, Jilin University, Changchun, China

**Keywords:** online popularity, interaction behavior, peer-to-peer lending, entrepreneurship, psychological perception, individual investors

## Abstract

Inappropriate social interactions of entrepreneurs can generate negative effects in the peer-to-peer lending market. To address this problem and assist peer-to-peer entrepreneurs in customizing their online interaction strategies, we used the cutting-edge cognitive-experiential self-system conceptual model and studied the relationship between peer-to-peer entrepreneurs’ interactions and financing levels. Online interactive information was categorized as emotional or cognitive, adding the moderator of entrepreneur popularity, and the effect of these interactions on individual investors was analyzed. We found that the entrepreneurs’ online interactive information affected psychological perception of entrepreneurs and their corresponding brand image. The interaction between popularity and interactive information types was significant. The findings imply that less popular entrepreneurs should engage in emotional interactions, while more popular entrepreneurs should choose cognitive interactions. Online interaction created comparative advantages in the financing activities of peer-to-peer companies. These results expand understanding of the psychological facets of the consumer–brand relationship in the digital world, and extend the current literature. This study also highlights key areas of learning and application for both practitioners and scholars of organizational psychology.

## Introduction

When studying entrepreneurship, the impact of social interaction must be considered (Aldrich and Zimmer, 1986). In recent years, the focus of academic research on the role and behavior of entrepreneurs has gradually shifted from internal managers to external social influencers. The social interaction of entrepreneurs is an important aspect of front-end behavior display, and has received extensive attention within academic and professional circles. Entrepreneurs’ positive social behaviors enhance the company’s legitimacy and goodwill (Daily and Schwenk, 1996). As leaders of a company, entrepreneurs’ personal behaviors and information they provide have a direct bearing on corporate image and value. For example, on September 6, 2018, the chairman and CEO of JD.com was arrested during a visit to the United States. JD’s stock price fell by 16% within 2 days, and the group’s market value evaporated by US$7.2 billion (Sohu, 2018). On September 10 of the same year, the chairman of the Alibaba Group announced his retirement plan. The stock price fell 3.7% on the same day, and US$15 billion of the group’s market value was lost (IFeng Finance, 2019). The CEO of Tesla was exposed as having smoked marijuana, which caused market concerns about the group’s prospects. That day, the Tesla stock price fell 6.3% (NetEase Tech, 2018). Business leaders can directly affect the value of the company, and a negative personal reputation may reduce the company’s legitimacy and goodwill. With the rapid development of the internet, entrepreneurs face ever-increasing intensity of online social interactions and frequency of information demanded. Etemad et al. (2010) wrote: “Relations on the internet are more explicit than those based on interpersonal and culturally or socially based relations. In fact, most of the online relations are of functional and professional nature, explicitly articulated, and do not lend themselves to interpersonal or cross-cultural misunderstandings or misinterpretations, whether favorable or not.” Aouadi et al. (2013) pointed out that Google search volume is a reliable proxy of investor attention, and a lot of investment information content is often available on the internet.

Compared with other industries, there are particularities in the peer-to-peer lending field (P2P). At the time of the study, China’s P2P lending industry was an investor’s market, with more demand for financing than supply of funds. Because P2P online loans require a large amount of social capital investment, this industry is attracting a large number of individual investors (Wang, 2018). It should be noted that, in this paper, we exclude institutional investors. In the field of P2P lending, the purpose of entrepreneurs when interacting online is not only to send information to those who need funds, but also to attract the attention of investors. Compared with consumption, investments pose higher risks. Investors make funds available without the exchange of products, services, or added value, and face the risk of losing their principal investment. China’s P2P lending market was a late entrant. Because of asymmetric information in the industry, lack of supervision, and inadequate platform risk control, many of the hazards in the industry have been realized in recent years. Some platform’s capital chains have ruptured, causing many investors to suffer losses, thus increasing the negative impact on the development of this industry (Zhang, 2013). In the field of P2P lending, the three factors of high industry risk, severe information asymmetry, and investor risk aversion have been compounded, leading to the individual investor’s preference for more reliable rather than higher-yield projects (Wang, 2015). Simultaneously, there are small- and medium-sized platform enterprises in China’s P2P industry (with a registered capital of less than 30 million yuan, or approximately US$ 4.29 million). Braune et al. (2016) pointed out that the financial efficiency of SMEs is low, with poor profit outlook for members in this industry cluster. These small- and medium-sized P2P enterprises are more dependent on their own comparative advantages (Song et al., 2018). Due to policy restrictions, P2P platforms cannot publish advertisements in the mainstream media, and it is difficult to relay reliable information to individuals (Yeo and Jun, 2020). Therefore, compared with other industries, the influence of business leaders is more prominent, and personal, social, and online interaction of entrepreneurs using P2P platforms has become an important comparative advantage. To what extent does the online popularity of entrepreneurs affect P2P lending platforms? What types of information released by entrepreneurs achieve superior results? These extremely practical problems have piqued the author’s research interest.

This paper is divided into several parts: literature review, theoretical framework, methodology, analysis and discussion of results, and conclusion. We also fully discuss the existing research, theoretical logic, data extraction, and measurement, analysis of results, and the conclusions drawn, offering several academic contributions and managerial implications. This paper innovatively takes the P2P lending industry as a research object and explores the impact of entrepreneurs’ online interactions on financing, through empirical research, which has associated practical implications. Additionally, the innovative inclusion of variables that enhance the popularity of entrepreneurs and their online interactive content, while analyzing their interaction and resultant impact on corporate financing, can provide a reference for further academic research.

## Literature Review

### P2P Lending

P2P lending refers to the use of internet platforms to complete transactions between the demanders and suppliers of funds, constituting small loans between individuals. P2P online lending originated in the United Kingdom, and then expanded to the United States, Germany, and other countries. Its typical model consists of an online credit company that provides a platform where both borrowers and qualified lenders can bid freely. In the traditional P2P model, the online loan platform only provides information-sharing activities—information on value identification and other services that facilitate the completion of transactions for both parties—but without directly participating in the loan value chain. There is a direct relationship between the borrower and the lender, and the online loan platform charges a service fee from the borrower and lender to maintain its operation. The modern P2P platform also has crowdfunding investment projects, together with wealth management products. In 2015, China’s online loan transaction volume exceeded one trillion yuan (US$0.14 billion), a year-on-year increase of 258.62%, and historical cumulative transactions were 16.31 billion yuan (US$2.33 billion) (China Economics, 2016). Likewise, with its unique role, P2P online lending has emerged on a large scale around the world and become a major supplement to traditional finance sources. Wang and Liao (2010) pointed out that this new form of borrowing reduced transaction costs and greatly expanded the coverage of financial services. Empirical research on P2P online lending found that, in terms of borrower characteristics, the main targeted users of P2P loans are short-term, low-value lenders who are rejected by traditional financial institutions because credit information cannot be transmitted accurately or because they cannot provide sufficient collateral. Most borrowers come from the working class with poor credit. Zhuang and Zhou (2015), by reviewing order statistics released by PPdai.com, found that, on average, loan amounts were 7371.795 yuan (US$1053.11); bids attracting investors to participate in the loan slip were 19.9148; the winning rate of the loan slip was 0.3252; the term was 6.5 months; and most P2P borrowers had a loan period of less than 1 year. Guo’s (2013) research concluded that users of P2P network loans are unevenly distributed in terms of region, age, education, and credit rating. You and Zhang (2010) believe that P2P online loans have filled the gap created due to the lack of services provided by financial institutions to small- and medium-sized enterprises and vulnerable groups in formal finance. The unsecured guarantee, self-service transaction model, short-distance transaction space, and new financial management options provided by P2P lending have met the financial needs of many small- and medium-sized customers, and play an active role in promoting independent entrepreneurship. The P2P platform can share interest rates with borrowers and lenders due to lower credit evaluation and operating costs, which is an effective way to serve small and micro businesses and realize inclusive financing (Song et al., 2014).

### Online Interaction

#### Overview of Online Interaction Vehicles

In 2006, Evan Williams, the founder of Twitter, first proposed the concept of microblogging. Gaonkar and Choudhury (2007) suggested a comprehensive definition, describing it as a multimedia blog that mainly focuses on the following four functional elements: wireless networks, information processing, space visualization, and mobile phone sensors. Subsequently, scholars defined micro-blogging from the perspective of information dissemination; the most cited definition, proposed by Kaplan and Haenlein (2010), is that of a micro-blog which is a technology-based internet exchange tool that allows users to create or exchange user-generated information content. In the past, people used the internet to simply consume “content,” such as reading online information, watching online videos, or purchasing products and services (Kietzmann et al., 2011). A micro-blog is a social media tool that uses technology to achieve communication, transactions, and relationship construction between users, and enables enterprises to gather consumers and create value together (Andzulis et al., 2012). People will also publish descriptions, opinions, and evaluations of brands and products on micro-blogs (Jansen et al., 2009). In addition, micro-blogging is an important platform to convey personal values. For example, in emergencies, micro-blog content has often indicated concern for the status of refugees, by suggesting rescue actions, while fostering commitment and cooperation (Smith, 2010). Users with higher sociability, extraversion, or emotional needs prefer to use Facebook, while those with higher cognitive needs tend to choose Twitter (Hughes et al., 2012). Unlike in Western countries, because of policy reasons, mainland Chinese netizens cannot use Facebook or Twitter. Instead, Weibo was launched by a Chinese company called Sina. Statistics show that the number of Chinese Weibo users in 2018 was 337 million, an increase of 34.56 million compared with 2017, accounting for 42.3% of the total number of internet users. Because of the lack of competition, Weibo has become China’s most used text self-media (AskCI, 2019).

#### Business Applications of Micro-Blogging

Because users often communicate with each other and share opinions about brands through micro-blogging, as an important “word-of-mouth” disseminator of resource data, it is used by companies to drive business forecasting. Asur and Huberman (2010) used consumer comments on movies on Twitter to predict movie box office hits. The study found that people’s attitudes to movies on micro-blogs were significantly positively correlated to movie box office revenues. Tayal and Komaragiri (2009) used micro-blogs and blog data to predict stock prices. Their results showed that the correlation coefficient between the predicted value of micro-blog data samples and the company’s real stock price was greater than 0.9, and the accuracy of micro-blog data prediction was significantly higher than that of blog data. By analyzing the content of consumers’ micro-blog information, enterprises may better understand the factors and changing trends that influence consumers’ evaluation of brands. Jansen et al. (2009) analyzed the semantic and attitudinal structure of 150,000 pieces of consumer micro-blog content containing brand information on Twitter, and found that micro-blog information contained a large number of consumer evaluations (both positive and negative) of different brands, as well as changing trend characteristics. Additionally, by using these data, companies collect information about competitors and predict their actions. Thus, consumers have adopted micro-blogs as an important and credible channel to obtain brand information and exchange brand opinions, which has created new opportunities for companies to establish consumer-brand relationships through these platforms. Enterprises can interact online with consumers *via* micro-blogs and influence their purchasing decisions and perceptions of brand image, while assisting them in solving their product-related problems (Hsu et al., 2010; Coyle et al., 2012).

#### Entrepreneurs’ Online Interaction on Micro-Blogs

Entrepreneurs publicly disseminate information, communicate, and interact with the public under their real-name identity in the micro-blog environment. Entrepreneurs experience three stages, i.e., “textualization,” “mediatization,” and “digitalization” in external communications (Girginova, 2013). From the earliest paper media, such as newspapers and magazines, to electronic media such as radio and television, and now to the digital communication methods of micro-blogging, entrepreneurs’ information has been disseminated at an ever-increasing pace to an expanding audience. In modern marketing practice, it has become a trend for entrepreneurs to identify themselves on the micro-blog platform and interact directly with the public. Scholars believe that their primary motivation is to influence and impact (potential) consumers and target groups (Fischer and Reuber, 2011; Hartzel et al., 2011; Huang et al., 2014). When entrepreneurs, as representatives of the enterprise and symbols of personalization (Mazur, 1999; Gaines-Ross, 2000), use micro-blogs to communicate directly with the public, their image is established as genuine and transparent in the minds of consumers (BrandFog, 2012). Micro-blogs thus deepen consumers’ awareness of entrepreneurship and brand image (Samuel, 2009), and foster mutual connections and relationships (Girginova, 2013). Research shows that 82% of consumers trust entrepreneurs who participate in online activities, while 77% indicate a purchasing preference for their products and services (Cruz and Mendelsohn, 2012). Girginova (2013) collected and analyzed 2086 tweets and interview records published by ten entrepreneurs and found that the micro-blog information released by entrepreneurs may generally be categorized as personal-related content or work-related content. This information will affect consumers’ perceptions of, and preferences for, the entrepreneurs’ image and branding. From the perspective of social interaction, Fischer and Reuber (2011) found that, compared with information devoid of emotion, emotion-rich information compels consumers to evaluate entrepreneur image and brand image more highly; relative to corporate-related information, entrepreneur-related information will elicit a more positive consumer evaluation of corporate image and branding.

Introductory and theoretical studies on micro-blogging have been conducted by enterprises and entrepreneurs. However, given that the online interaction goals and communication characteristics of entrepreneurs in the P2P lending field are different from those in traditional industries, their financing, rather than sales characteristics, have prevented the application of existing entrepreneur interaction strategies to the P2P lending field; this highlights the importance of the current discussion, as undertaken in our research, which supplements the relevant literature on entrepreneurial behavior theory and provides practical implications.

### Comparative Advantages of Entrepreneurs’ Social Interaction

The intentional presentation or unintentional exposure of entrepreneur information to the public has a direct impact on their image shaping. Examples include entrepreneurs’ advertising endorsements, external communications, charitable acts, and negative behaviors, among others. First, it is commonplace for entrepreneurs to offer advertising endorsements to great effect. Friedman et al. (1976) took a virtual brand of sparkling wine as an example, and used movie stars, typical consumers, experts, and entrepreneurs as spokespersons in the advertisements to test the endorsement effect. The study found that compared with other types of spokespersons, entrepreneurs were rated most highly by consumers on image evaluation and product purchase intentions, and their findings were later supported by Freiden (1984). Second, for entrepreneurs, public speaking (external communication) is an important way to shape their image. Their personal charm can be successfully demonstrated by explaining the vision, language, and emotion of their brand (House and Shamir, 1993). Clearly, the unique communication styles of entrepreneurs will cause consumers to develop special image associations. Third, the charitable behavior of entrepreneurs, especially their actions taken in the face of sudden catastrophic events, can earn the public’s goodwill (Wang, 2011; Huang et al., 2012; Guo et al., 2020).

The brand personality of an enterprise is critical (Cai and Mo, 2019). First, entrepreneurs’ social interactions can affect corporate branding (Nazir et al., 2020). When interacting with the public, the entrepreneurs’ personality and voice represent and shape those of the entire organization (Garbett, 1988; Gray and Balmer, 1988). Cognitive balance theory proposes that if consumers have a positive impression of entrepreneurs, this will be reflected in their attitudes to the brands and products of the entrepreneurs’ company, as indicated by numerous empirical studies (He and Wang, 2005). Moreover, when consumers strongly associate entrepreneurs with corporate brands, this has significant positive impact on corporate brand image (Huang et al., 2010).

Second, entrepreneurs’ social interaction affects corporate performance (Agle et al., 2006). Effective social interaction by entrepreneurs has a significant impact on company stock prices (Tosi et al., 2004). Leadership styles also have a significant impact on the company’s financial performance (Yuki, 2008). Nguyen-Dang (2005) conducted a study on Fortune 500 companies from 1992 to 2002, and the results indicated that level of media exposure to CEOs had a significant positive impact on company performance, with positive reports having a greater impact.

Third, the social interaction of entrepreneurs affects their key role in securing access to external resources from investors, including financial, material, and intangible capital (Starr and MacMillan, 1990). Securing external resources not only enables companies to undertake development opportunities, but also greatly enhances their prospects of survival and profitability (Brush et al., 2001). Flynn and Staw’s (2004) research shows that entrepreneurs who enhance their personal charm by actively interacting with the public ensure that their companies obtain additional external resources. Charismatic entrepreneurs are better able to attract attention and commitment of external investors, especially when the macroeconomic situation is poor or the company encounters a crisis. Treadway et al. (2009) found that media attention and entrepreneurs’ political skills will affect their ability to convert their reputation into goodwill, enhancing their personal success and the financing efficiency of their companies. Shaping the public image of entrepreneurs means expanding their personal popularity and ultimately assisting companies in gaining the favor of investors.

In summary, there are still two areas of extant research that require improvement. First, the analysis of entrepreneurs’ social interaction is one-way rather than two-way. In the past, scholars have mostly discussed passive interaction behaviors, wherein, rather than being actively shared with the public, most information about entrepreneurs was passively exposed by the media. This is inconsistent with the current two-way interaction model between entrepreneurs and the public. The second is that the communication media in extant studies, including TV, radio, and printed media, were generally outdated. Studies of the impact of entrepreneurs’ active communication behaviors on consumers and investors in the context of new digital media were lacking. In the online environment in particular, entrepreneurs can achieve direct dialogue, self-presentation, and disclosure with the public: this requires further study. This research was thus performed from the perspective of online interaction.

## Theoretical Framework

The research question established in the literature review is as follows: how do the online interactions of entrepreneurs affect public perception of their image and the financing level of P2P platforms? The theoretical model and research hypotheses of this study are constructed considering relevant research concepts, namely reputation theory, and cognitive-experiential self-system theory.

### Individual Reputation Theory

Fama (1970) proposed the reputation theory and applied it in many fields of research and business administration. With the continual advancement of internet technologies, many consumers purchase goods and services from online companies. Consumers are cautious in accepting new processes inherent in internet-based companies, and reputation is a key factor for them. As components of the internet financial industry, P2P online lending companies are no exception. Therefore, many scholars have begun to pay attention to the reputation of online entrepreneurs. Moreover, in an online environment, reputation formation, and accumulation is rapid, the resultant flow of this information is efficient, and the generation of information networks is effective. Koufaris and Hampton-Sosa (2004) define the reputation of internet-based entrepreneurs as the public’s trust in, and consumers’ attention to, these company leaders. Pavlou (2002) indicates that online entrepreneurs are concerned that customer’s dissatisfaction will affect their reputation; hence, they pay more attention to behaviors that may adversely affect this. Jarvenpaa et al. (2000) found that there is a positive correlation between the personal reputation of internet-based entrepreneurs and customer trust; that is, entrepreneurs with a positive reputation can help alleviate the negative impressions formed by customers.

The problem of adverse selection due to information asymmetry may cause the market to collapse. In the P2P online lending market, one way to overcome this problem is for the P2P lending platform to assume guarantee responsibility by establishing their own reputation through repeated lending transactions. Through high-credit transaction matching, investors can evaluate the stability and reliability of the platform and make secondary investments. At the same time, through the actions of investors, a P2P platform with a strong reputation may create arbitrage opportunities and benefit from more favorable interest rates. Therefore, the “reputation model” in this market should be based on dynamic analyses.

There are two types of P2P platforms in the network lending market: high-quality and sub-optimal, which are labeled as platform 1 and platform 2. The financing cost corresponding to the high-quality platform is r_1_, and the financing cost corresponding to the sub-optimal platform is r_2_, where r_2_ > r_1_ > 0; that is, the financing cost corresponding to the high-quality platform is lower than that corresponding to the sub-optimal platform. We suppose that the P2P lending platform has financing needs, and the income from the project invested or lent is B, and the investor’s reputation for the platform is s. Further, we assume that the investor’s utility function is L = Ns-B, where *N* = 1 means that the high-quality platform gets financing; and *N* = 0 means that the low-quality platform gets financing. Then, the utility function of platform 1 is b_1_ = B-r_1_, and the utility function of platform 2 is b_2_ = B-r_2_, where b_2_ < b_1_. The utility of investing funds in high-quality platforms is s-B, and the utility of investing in sub-optimal platforms is -B. The social welfare generated by investors who give capital to high-quality platforms is *F* = (B-r_1_)+(B-s) = s-r_1_. If s is greater than r_1_, the social welfare is greater than zero. If the investor invests the funds in a sub-optimal platform project, the social welfare is F = (B-r_1_)+(-B) = -r_1_, which is less than 0, indicating that the overall benefits of online lending are impaired.

Assuming that the high-quality platform has undertaken online lending k-1 times, and now it is the kth time, assuming the discount factor of time is a (a < 1), the utility obtained after engaging in several lending transactions is:


$$\left(B-r_{1}\right)\,a\,\left(B-r_{1}\right)\,\ldots \,\ldots \,a_{k-1}\,\left(B-r_{1}\right)=\frac{\left(1-a_{k}\right)\,\left(B-r_{1}\right)}{\left(1-a\right)}$$

If platform 1 deteriorates and drops in quality to become a sub-optimal platform, then the current gain is B-r_2_. So, the platform will continue to maintain a good reputation:


$$\frac{\left(1-a_{k}\right)\,\left(B-r_{1}\right)}{\left(1-a\right)}>B-r_{2}$$

If the number of loans becomes infinite, that is, the value of k tends to infinity, the above formula can be transformed into:


$$\frac{\left(B-r_{1}\right)}{\left(1-a\right)}>B-r_{2}$$

Rearranged as:


$$\text{B}>{\text{r}}_{1}+\left(\frac{1}{\text{a}}-1\right)\,\left({\text{r}}_{1}-{\text{r}}_{2}\right)$$

The part of the formula that is less than the borrowing cost r_1_, which is [(1/a)-1](r_1_-r_2_), can be regarded as the “reputation rent” of the P2P platform—that is, the borrower’s utility satisfaction obtained from its reputation. It can also be found from this equation that if the borrower’s borrowing strategy continues to operate, from a probabilistic point of view, the borrower is unlikely to risk opportunism. The above helps to increase buyer loyalty (Cassia and Magno, 2012).

### Cognitive-Experiential Self-System Theory

#### Types of Online Interactive Information Used by Entrepreneurs

Since the 1990s, the changing economic environment has increasingly affected business operations, and it has become necessary for companies to adopt innovations and other means to protect their competitiveness (Geerguri-Rashiti et al., 2017; Ramadani et al., 2017). Scholars in the field of social psychology believe that entrepreneurs’ image is a competitive advantage for companies, and have conducted numerous studies and explored mechanisms for evaluating the attitudes of consumers. Among them, the cognitive-experiential self-system theory is a relatively representative theoretical achievement. It has been widely used in advertising research, consumer behavior, and other marketing fields. The model suggests that when people are processing information, there may be two sets of systems that function independently, namely, the cognitive and emotional systems. For a given stimulus, without additional cognitive coding, people’s emotional system will take the lead in responding. Cognitive systems tend to respond more slowly and require longer processing times (Epstein, 1994).

Emotional systems refer to self-cultivation and self-improvement in interpersonal communication to attain perfection, in the form of aesthetic sublimation, moral perfection, or deepening of religious belief. This aspect of cognition can manifest as the transmission of personal values, such as charitable behavior, the release of positive energy, or the transfer of social evaluations. The cognitive system emphasizes the relentless pursuit of knowledge, and applies the acquired knowledge combined *via* scientific methods to all aspects of people’s lives, such as political and economic considerations, to obtain practical benefit. In the commercial field, this is mostly expressed as the promotion of enterprises and products. Emotional processing refers to a person’s communication within the scope of the family and private interactions. Cognitive processing refers to communicating with others in the public domain (Kim et al., 2012). The cognitive and emotional systems, also known as the cool and hot systems, deal with information differently. The cool system is responsible for complex characterization, analysis, and thinking, involving time, space, and scenario analysis. Information processing speed is slow. The hot system is mainly responsible for the rapid emotional response to processing of information. It often produces an intuitive and automated response, and the processing speed is relatively fast (Metcalfe and Mischel, 1999). Both systems can independently influence the persuasive effect of information (Rosselli et al., 1995). Based on the above, different types of online interactive information of entrepreneurs will lead to different responses from consumers.

#### Entrepreneur Popularity and Interactive Information Types

Entrepreneur popularity refers to the public’s knowledge of entrepreneurs, that is, how well known they are. The amount of information available to consumers about entrepreneurs depends on the entrepreneurs’ popularity, resulting in alternative knowledge bases for consumers to judge and evaluate entrepreneurs (Briley and Aaker, 2006). When the popularity of entrepreneurs is low, consumers obtain less information from the cognitive system and more from the emotional system. Extant research shows that prominent celebrity endorsements can attract consumers’ attention and increase their willingness to process information (MacInnis et al., 1991) by prompting consumers to remember product features and advertising content. Therefore, compared with high-popularity entrepreneurs, consumers pay less attention to micro-blog information of low-popularity entrepreneurs, and their motivation to process complex information is weaker (Briley and Aaker, 2006).

### Theoretical Derivation and Hypotheses

According to the previous theoretical analysis, in the context of the P2P lending industry and differing levels of popularity among entrepreneurs, this article attempts to analyze what kind of online, interactive information—whether emotional or cognitive—investors prefer. We make the following theoretical derivations and hypotheses.

#### Entrepreneurs’ Online Interactive Behavior and Investor Feedback

Entrepreneur image is the overall perception of entrepreneurs by individuals or the public, which is affected by the words and behaviors of entrepreneurs (Gao et al., 2020). According to the theory of social information processing, people make judgments and evaluations of others through available information channels (Walther, 1992). Information dissemination through online social media has the characteristics of immediacy, wide radiation range, and fast speed (Mujahid and Mubarik, 2021), providing entrepreneurs with a platform for direct interaction and communication with consumers (Tafesse and Korneliussen, 2021). Compared with other self-display methods (news releases, behavioral exposures, etc.), entrepreneurs’ online interactive information is easier to retain and trace back, and thus easier for consumers to obtain, enabling them to directly respond to, participate in, and interact with this information (Girginova, 2013). Therefore, the online interactive information of entrepreneurs serves as an important clue for the public to evaluate entrepreneurs and affects the image of entrepreneurs in the minds of the public (Olanrewaju et al., 2018).

When entrepreneurs spread information online, investors or consumers will directly obtain internal clues to evaluate entrepreneurs (Choi, 2021). According to impression management theory, people often try to influence the process of others’ impression formation. In the process of interacting with others, people manage others’ impressions of themselves by conveying favorable identity characteristics, with the goal of obtaining the desired results in the interaction (Abbas et al., 2019). In this context, entrepreneurs try to shape and spread a positive image of themselves through online social platforms, because a positive entrepreneur image can be used as a powerful signal to influence the audience (such as consumers and investors) and thus promote corporate brand performance (Shu et al., 2018). Through “emotional” information, investors can interpret the entrepreneurs’ behavior. Such information reflects the entrepreneurs’ morality and enhances the investor’s empathy and psychological identity (Metzker et al., 2021). In comparison, through cognitive information, consumers can assess how the entrepreneurs undertake achievements by carrying out creative activities, which reflect the ability of the entrepreneur (Yuan and Peluso, 2019). Through the interaction of emotional and cognitive information, entrepreneurs try to convey a positive image to investors and the public. Therefore, we propose the first hypothesis:

*H_1_: During their online interactions, entrepreneurs will get positive feedback from investors regardless of whether they convey cognitive or emotional information.*

#### Entrepreneur Popularity and Investor Feedback

Entrepreneur popularity refers to the degree of public awareness of individual entrepreneurs. When the popularity of entrepreneurs is different, the amount of entrepreneur information acquired and possessed by investors is different, leading to different knowledge bases for investors to judge and evaluate entrepreneurs (Kašperová, 2021). When the entrepreneurs’ reputation is low (high), the consumer or investor’s personal knowledge obtains less (more) entrepreneur information. Studies have shown that information containing celebrity clues attract consumers’ attention and increase information processing willingness more strongly (Albert et al., 2017; Liu and Liu, 2019; Zhu et al., 2020). For example, advertisements endorsed by celebrities can prompt consumers to remember product features and advertising content. Therefore, the public is less willing to pay attention to the microblog information of low-popularity entrepreneurs than that of high-popularity entrepreneurs, and the motivation for information processing is weaker for the former. In the case of insufficient information processing motivation, the public’s cultural knowledge clues will become an important aspect that affects their judgment and decision-making (Briley and Aaker, 2006). The so-called cultural knowledge includes beliefs, values, attitudes, and other concepts used to explain and guide behavior, shared by members of the community (Torelli et al., 2021). Cultural knowledge clues are the emotional information mentioned in this article, which is highly available and can be automatically activated at an unconscious level (Stanley et al., 2021). Therefore, we propose the second hypothesis:

*H_2_: The popularity of entrepreneurs and online interactive information have a positive effect on investor feedback.*

#### Types of Entrepreneurs’ Interactive Information and Investor Feedback

When consumers are less motivated to process information, how do people use cultural knowledge clues to evaluate entrepreneurs? Briley and Aaker (2006) suggest that when entrepreneurs are less well-known, the public is more likely to be influenced by cultural knowledge clues. The public is more likely to pay attention to the ethics of entrepreneurs rather than how professional they are. Relevant emotional information can reveal the entrepreneurs’ personality and credit level, and it is more likely to be highly praised and psychologically recognized by the public. Therefore, we propose the third hypothesis:

*H_3_: For less-popular entrepreneurs, investors respond more positively to the presentation of personal values and other emotional information.*

However, when entrepreneurs are well-known, the public has more information about entrepreneurs. Compared with less popular entrepreneurs, the interactive information of highly popular entrepreneurs will attract more public attention and trigger more information processing (MacInnis et al., 1991). According to Weber’s (1966) social stratification theory, personal prestige, power, and wealth signify the level of different individuals in social stratification. Compared with less popular entrepreneurs, people are more likely to associate highly popular entrepreneurs with high personal reputation, high power, and wealth, and therefore are more likely to believe that they have a higher social status (Bonner et al., 2021). Consumers pay more attention to people with high social status in terms of how they succeed and obtain achievements, i.e., rational cognitive information. Therefore, we propose the fourth hypothesis:

*H_4_: For more-popular entrepreneurs, investors respond more positively to the presentation of product details and other cognitive information.*

**FIGURE 1:**
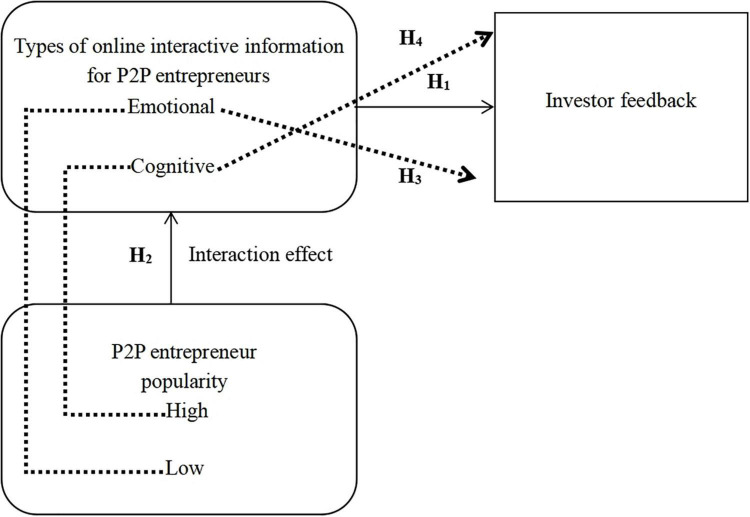
Theoretical logic framework.

Through the above theoretical derivation and argumentation, a theoretical framework, as shown in Figure 1, is proposed.

## Methodology

### Variables

#### Independent Variable: Types of Online Interactive Information

We encoded and classified all Weibo (the most popular micro-blog platform in China) data from the previous year. Among these data, we identified entrepreneurs’ emotional online interactive information, indicating self-expression or relationship processing with others; this included information on their hobbies, family and life experiences, and opinions on public social events (Zhu, 2014). Cognitive information is defined as information about the practical affairs of entrepreneurs in their professional roles. These include their entrepreneurial management philosophy, insights into the development of industries and enterprises, daily management of work, and product updates (Zauberman et al., 2006).

Referring to the method of Bakshy et al. (2011), we invited three independent adjudicators to encode the content of each Weibo message, distinguishing between “emotional” and “cognitive” information. Before coding, we explained the related concepts to ensure consistency, independent assessment, and thorough understanding of concepts. Values were then assigned to the information content (based on the ratio of the two different types of information published by each entrepreneur), with a value range of 1–9. The closer to 1 the values were, the greater the proportion of “emotional” information they represented; the closer to 9, the greater the proportion of “cognitive” information. Finally, we take the average value of the three independent adjudicators (Berger and Iyengar, 2013) as the basis for measuring the type of information (TOI) in Weibo.

#### The Moderating Effect of Entrepreneurs’ Online Popularity

In the context of Weibo, the number of fans reflects the entrepreneurs’ influence, popularity, and the overall attitude of the audience (Cha et al., 2010). Zhu (2014) used the number of Weibo fans to measure the online popularity of entrepreneurs, and the median of the total sample to distinguish between high and low popularity. We referred to her method and recorded high popularity as 1 and low popularity as 0. Because of the limited sample period and the relatively static online popularity of entrepreneurs, we referred to the method of Jin et al. (2011) and set a recent observation date to determine the number of fans. Popularity was recorded as POP.

#### Dependent Variable: Lending Amount per Capita

Hoffmann et al. (2015) and Thomas et al. (2021) defined the perception and feedback of investors as the degree of investor recognition of investment projects. Scholars such as Richardson et al. (2012), Autore and Kovacs (2014), and Liu (2019) have used the amount of investment to quantify the degree of recognition. On the basis of the above, we carried out per capita corrections on the variables. Our method recorded the per capita investment amount per investor on the P2P platform as an indicator of their recognition of the P2P platform, which was recorded as LPC.

#### Control Variables

Liu et al. (2002) and Zhu (2014) found that the length of time a user spends on social networks and the frequency of posting information will significantly affect others’ impressions of them and the effectiveness of information transmission. The relationship between these two variables and the dependent variables in this article has been empirically tested. They are not experimental variables that we found necessary to investigate in this research. Therefore, we take the time span of entrepreneurs’ online interaction and the frequency of information release as control variables. Referring to the method of Liu et al. (2002), elapsed time, being the number of months for which entrepreneurs’ data was retrieved from Weibo, was denoted by TIM. The weekly number of micro-blogs published during the period was recorded as NUM.

### Data

Samples are generated from 252 P2P lending companies operating normally in mainland China. The filter conditions (and eligible candidates for each) are: a. As of the cut-off date, the company has been in normal operation for at least 5 years (from 252 number of samples to 88); b. There has been complete disclosure of enterprise-related data (from 88 number of samples to 69); c. The paid-up registered capital of the enterprise is more than 50 million RMB or approximately US$7.14 million (from 69 number of samples to 57); d. Entrepreneurs have registered on Weibo and are in an active state, that is, there are no fewer than 2 reposts or original posts per month (from 57 number of samples to 43). Details are shown in Table 1.

**TABLE 1:** P2P lending platform sample statistics.

Number	Enterprises	Entrepreneurs	Registered capital (million RMB)	Average loan duration (days)	Average interest rate
1	Renrendai	YANG	2,000	244	10.20%
2	Niwodai	YAN	550	360	10.80%
3	Eloancn	ZHANG	1,000	360	10.24%
4	5aitou	LIU	535	360	9.60%
5	PPmoney	CHEN	500	381	9.24%
6	Guangxindai	ZHANG	500	360	11%
7	Yooli	ZHANG	1,000	578	11.58%
8	Xiaoniu	PENG	103	360	8%
9	XSjinfu	Yuan	100	352	11%
10	HYJF	LIU	500	182	7.11%
11	Lending51	ZHANG	500	180	11%
12	Gxyclub	LIN	200	304	9.50%
13	91wangcai	XU	60	30	6%
14	Iqianjin	DONG	1,000	304	12.83%
15	Hurbao	MENG	60	100	10.33%
16	Juzilicai	XIAO	510	477	11%
17	Xywj	TANG	500	288	6.59%
18	Huilc	GUO	50	210	11.30%
19	Jrjc	ZHANG	66	180	9%
20	XSPH	ZHENG	111	90	8%
21	CGN	LUO	50	180	7.50%
22	Souyidai	ZHOU	300	395	8.55%
23	BJDP2P	WEN	142	103	7.28%
24	FYJF	LI	50.4	65	5.56%
25	51rzy	ZHONG	100	90	9%
26	NNb	LIN	100	730	12%
27	PHLC	LI	50	177	9.17%
28	YYfax	WU	70.4	720	8.20%
29	Qianlindai	HU	100	90	9%
30	HYR	SONG	500	785	11.83%
31	Niuyan	LUO	118	91	8.52%
32	Myerong	LIN	100	1,080	11%
33	TGJR	LI	50	180	9%
34	CCD	LIU	50	164	6.82%
35	WSD	Zhang	50	90	10%
36	GLJR	YANG	50	180	12%
37	XWJR	CHEN	50	95	5.60%
38	BGNC	ZHENG	120	462	7.44%
39	HJB	PAN	50	53	12%
40	I2p	CHEN	100	30	8%
41	JPB	GE	100	90	9%
42	CTZR	ZHONG	66.15	60	12%
43	ZYXR	LUO	52.63	180	10%
**Mean**			**285**	**274**	**9.39%**

It can be seen from Table 1 that the average registered capital of the P2P platform selected is 285 million RMB (US$40.71 million), the average borrowing period is 274 days, and the average interest rate is 9.39%. The above data approximate the average values for China’s P2P lending industry, indicating that the sample selected is representative. In terms of methodology, this article focuses on the research methods of Dana and Dana (2005). The above data comes from the 01 Caijing database.^1^ The entrepreneurs’ micro-blog data were manually collected and sorted from Sina Weibo,^2^ and the time range is 1 year (from June 1, 2019 to June 1, 2020). The data processing tool is SPSS 25.0.

### Model

According to the previous theoretical analysis and research assumptions, this article builds a regression model with effects on interaction, as follows:


$$\text{LPC}=\,\alpha +\beta_{1}\times \text{TOI}+\beta_{2}\times \text{POP}+\beta_{3}\times \text{TOI}\times \text{POP}+\beta_{4}\times \text{Cont}+\varepsilon$$

## Analysis and Discussion of Results

### Descriptive Analysis

Descriptive statistics are shown in Table 2. Forty-three entrepreneurs published 43,963 Weibo posts during the research period, and the average number of Weibo messages posted by each entrepreneur per week was approximately 21. The average number of fans is 614,400. From the perspective of information content, entrepreneurs on P2P lending platforms are more inclined to publish emotional information than cognitive information. The average time that sample entrepreneurs have used Weibo is 31.4 months.

**TABLE 2:** Descriptive analysis.

Title	Min.	Max.	Mean
LPC (thousand RMB)	0.19	78.8	8.5
TOI	1	9	4.07
POP (thousand people)	24.3	3757.1	614.4
NUM	2	299	21.3
TIM	14	49	31.4

### Regression Analysis

The multiple regression method is used, where independent variable, moderator, the interaction term of the independent variable and the moderator, and the control variable are included gradually. To test the interaction term, we performed mean centralization of all continuous variables to reduce the problem of multicollinearity. The average value of the processed variable samples is zero, and the sample distribution remains unchanged. The diagnosis of multicollinearity shows that all VIF values are lower than 5, which indicates that the problem of multicollinearity is negligible and has no effect on the hypothesis test results. The specific results are shown in Table 3.

**TABLE 3:** Regression analysis.

	Model 1		Model 2	
Variables	Coefficient	*t*-value	Coefficient	*t*-value
Constant	0.126	10.573**	0.128	10.321**
TOI	–0.154	–4.561**	–0.186	–3.958**
POP	–0.017	–5.903**	–0.016	–6.362**
TOI*POP	0.033	4.909**	0.034	5.069**
TIM			0.027	1.042
NUM			0.036	3.051**
Adjusted *R*^2^	0.662		0.679	
Prob (F-statistic)	0.000**		0.000**	

**Represents significance at the 5% level.

First, we analyze the results for model one. From the given equation, it is evident that POP is a 0/1 variable. When the popularity of entrepreneurs is low, that is, POP = 0, β_1_ represents the influence coefficient of online interactive information on the financing level of P2P platforms. Table 3 shows that β_1_ < 0, and the test is significant at a 5% level. Therefore, H_3_ is accepted, that is, when the entrepreneurs’ popularity is low, TOI and LPC have a significant negative relationship. The greater the amount of emotional information reflected in the entrepreneurs’ online interaction, the better the investor’s feedback will be. This is inconsistent with most existing research results. The research conclusions of many scholars have shown that entrepreneurs with low popularity play little role in social networking. Both MacInnis et al. (1991) and Zhai (1999) pointed out that the public is less willing to pay attention to the microblog information of low-popularity entrepreneurs, and their motivation for information processing is weaker. This may be mainly attributed to the classification of the online interactive information of entrepreneurs, which is one of the innovative points of this article. Studies that do not consider information classification and interaction effects may report different results. Despite this, this result still holds theoretical logic. Based on the cognitive-experiential self-system, emotional information will not be ignored, but will be treated intuitively. When entrepreneurs were less well-known, the public was also influenced by cultural knowledge clues (Briley and Aaker, 2006). In addition, there is another point that needs special attention. In China, because of the influence of traditional culture and values, people tend to pay more attention to the morality of the other party in their interactions, even in business. This kind of business partnership established by trust can sometimes extend beyond factors such as price and profit, the difference being that western countries have a long history of capitalist development and pay more attention to non-subjective factors such as efficiency, profit, and spirit of contract in their relationships, and the attributes of economic man are more prominent (Liao, 2004). Therefore, this research result is also, to an extent, a product of the unique Chinese cultural background.

When the entrepreneurs’ popularity is high, that is, POP = 1, (β_1_+β_3_) represents the impact of the entrepreneurs’ online interaction type on investor feedback. Table 3 shows that (β_1_+β_3_) > 0, and both β_1_ and β_3_ are significant at a 5% level. Therefore, both H_2_ and H_4_ are accepted, that is, the popularity of entrepreneurs and the type of online interactive information have significant effects on investor feedback. When the popularity of entrepreneurs is high, TOI and LPC have a significant positive correlation. The more cognitive interactions entrepreneurs make, the better the investor’s feedback will be. This is consistent with the existing literature, such as BrandFog (2012), Cruz and Mendelsohn (2012), and Bonner et al. (2021). The presentation of product details and other cognitive information needs to be processed by the cold system, as described earlier in this paper. The slow speed of information processing requires the high popularity of entrepreneurs to mobilize investors’ enthusiasm for information processing.

In model two, we added relevant control variables, and the model’s goodness of fit was improved. The correlation and interaction relationships between the respective independent variables and dependent variables was unchanged, and the significance level was consistent with that for model one. This shows the robustness of the model, while verifying H_2_, H_3_, and H_4_. Both models β_1_ and β_3_ are significant at the 5% level and (β_1_+β_3_) is greater than 0; therefore, H_1_ is verified. During the online interaction of entrepreneurs, regardless of whether they convey cognitive information or emotional information, they will get positive feedback from investors. Although other scholars have reached similar conclusions (Larson, 2009; Zhang and Wang, 2016; Tian, 2017), it is worth noting that they did not classify information types, which is one of the innovations of this paper. All research hypotheses in this article are accepted. Most of them are similar to existing research (Shu et al., 2018; Abbas et al., 2019; Choi, 2021) and supported by theories such as individual reputation theory.

In terms of control variables, the number of online interactions of entrepreneurs is positively correlated with investor feedback. This shows that a larger number of online interactions leads to a higher probability that investors will participate in the company’s business. This is consistent with previous studies. Lemay et al. (2020) found that there is a positive correlation between the frequency of online social networking and social quality. Candel et al. (2021) suggested that the frequency of interaction is an important indicator of social relationship satisfaction. The length of time that entrepreneurs use Weibo has no relationship with independent variables. There was a reason for this, i.e., people use Weibo mainly through dynamic push. In this process, the time and content of the release are more important than the frequency of the release. The value of posting on Weibo on weekends may be greater than that of posting late at night on a working day (Kumar and Sinha, 2016).

## Conclusion

The purpose of this research was to investigate the extent to which the online popularity of entrepreneurs affects P2P lending platforms and what types of information released by entrepreneurs achieve superior results. In addition, we sought to assist peer-to-peer entrepreneurs in customizing their online interaction strategies from the perspective of investor psychological perception. We examined the related literature themes, such as online interaction, entrepreneur impressions, and other relevant issues, at both theoretical and practical levels, focusing on the area of P2P lending. On the basis of current literature and theory, we proposed an innovative classification standard for online interactive information of entrepreneurs and discussed its impact on financing. We used the cutting-edge cognitive-experiential self-system conceptual model to divide the types of online interaction information into emotional and cognitive types, and examined the problems related to interactions. Supported by the reputation theory and cognitive-experiential self-system theory, the following conclusions were drawn through theoretical deduction and empirical research.

First, we found that on P2P platforms, entrepreneurs act as social influencers and transmitters of reliable corporate information, and their online interactive content has a significant impact on public investors. Within the theoretical framework, this article classified this interactive content as emotional or cognitive information, and clarifies the impact of these two types of content on the public. Through empirical research, it was verified that entrepreneurs can produce positive effects regardless of whether they present emotional or cognitive information. Second, with the help of reputation theory, we included the popularity of entrepreneurs in the scope of observation and used it as a moderating variable to consider its effect on interactions. We were surprised to find that the popularity of entrepreneurs can be added to the model as a moderating variable, and the interaction between it and the TOI is significant. When the popularity of entrepreneurs is low, they can also get a positive response from investors by publishing more emotional information online; on the other hand, when the popularity of entrepreneurs is high, cognitive information release is the better choice. The above research results show that the popularity of entrepreneurs and online interaction behaviors do have a significant impact on public feedback. These findings are consistent with research conducted by other academics (Briley and Aaker, 2006; Albert et al., 2017; Olanrewaju et al., 2018).

With the support of theory, through the discovery of the interaction between different variables, this paper expands the research horizon of the psychological facets of the consumer-brand relationship in the digital world, and extends the current literature and theory. In addition, there are particularities in the P2P lending industry. We have refined the research areas and variables, while proposing theoretical models and research equations. The empirical test in this research identified the interaction effect between entrepreneurs’ popularity and online interactions and drew corresponding conclusions based on their level of popularity, which can provide a clear quantitative reference for subsequent research. This article further indicated that online interaction of entrepreneurs is not a straightforward, personal behavior, but requires careful planning by enterprises and entrepreneurs as part of long-term enterprise strategy implementation to provide information to investors. It can thus positively impact P2P companies, improve their financing levels, and provide comparative advantage through the optimization of corporate strategy. Through its strong theoretical foundation, this study highlights key areas of learning and application for both practitioners and scholars of organizational psychology.

We have designed a strategy for online interaction among P2P entrepreneurs. Entrepreneurs should clearly identify their online, interactive content characteristics, and combine their own popularity in adopting appropriate and effective interactive strategies. Low levels of popularity require a focus on the use of emotional content to influence the audience, such as sharing daily life experiences, life philosophy, views on public events, and charitable behavior. Well-known entrepreneurs should focus on the promotion of products and corporate information, including sharing business, and management insights, insights on development, and successful experiences of the enterprise and industry, so as to minimize the transmission of emotional information. This article further indicates that the online interaction of entrepreneurs is not a straightforward, personal behavior, but requires careful planning by enterprises and entrepreneurs, as part of a long-term enterprise strategy implementation to bring information to investors. It can thus positively impact P2P companies and improve their financing levels, and provide comparative advance through the optimization of corporate strategy (Dana et al., 2008). Relevant empirical research conclusions can also be considered in the selection of entrepreneurs’ online interaction strategies.

This article does not consider how the process of studying entrepreneurs’ online interaction, may be magnified by situational factors such as major emergencies or new product launches. Different situations, such as the outbreak of negative news or a crisis in corporate public relations, will affect the impact of online interaction by entrepreneurs. Therefore, future research should focus on situation theory to explore the impact of the corporate life cycle and special contexts on online interactions. Situational variables may play a moderator role, and some may also be considered as independent variables, thereby enriching and improving the research process.

## Data Availability Statement

The raw data supporting the conclusions of this article will be made available by the authors, without undue reservation.

## Author Contributions

JA and HD were responsible for writing the initial draft of the manuscript and putting forward the main propositions. HD was responsible for further modification and improvement of the manuscript. GL was responsible for reviewing and editing the manuscript. All authors contributed to manuscript revision, and read and approved the submitted version.

## Conflict of Interest

The authors declare that the research was conducted in the absence of any commercial or financial relationships that could be construed as a potential conflict of interest.

## Publisher’s Note

All claims expressed in this article are solely those of the authors and do not necessarily represent those of their affiliated organizations, or those of the publisher, the editors and the reviewers. Any product that may be evaluated in this article, or claim that may be made by its manufacturer, is not guaranteed or endorsed by the publisher.

## Footnotes

1
https://www.01caijing.com/finds/index.htm

2
https://weibo.com/
